# Inhibitory Activity of Yokukansankachimpihange against Nerve Growth Factor-Induced Neurite Growth in Cultured Rat Dorsal Root Ganglion Neurons

**DOI:** 10.3390/molecules200814959

**Published:** 2015-08-14

**Authors:** Chiaki Murayama, Shimpei Watanabe, Motokazu Nakamura, Hisayoshi Norimoto

**Affiliations:** 1 Kampo Research Laboratories, Kracie Pharma, Ltd., Kanebo-machi 3-1, Takaoka, Toyama 933-0856, Japan; E-Mails: murayama_chiaki@phm.kracie.co.jp (C.M.); shimpei.watanabe@kracie.co.jp (S.W.); 2 Nakamura Skin Care Clinic, Kitashirocho 2-3-24, Joetsu, Niigata 943-0824, Japan; E-Mail: gen.9963@gmail.com

**Keywords:** yokukansankachimpihange, atopic dermatitis, nerve growth factor, dorsal root ganglion neurons

## Abstract

Chronic pruritus is a major and distressing symptom of many cutaneous diseases, however, the treatment remains a challenge in the clinic. The traditional Chinese-Japanese medicine (Kampo medicine) is a conservative and increasingly popular approach to treat chronic pruritus for both patients and medical providers. Yokukansankachimpihange (YKH), a Kampo formula has been demonstrated to be effective in the treatment of itching of atopic dermatitis in Japan although its pharmacological mechanism is unknown clearly. In an attempt to clarify its pharmacological actions, in this study, we focused on the inhibitory activity of YKH against neurite growth induced with nerve growth factor (NGF) in cultured rat dorsal root ganglion (DRG) neurons because epidermal hyperinnervation is deeply related to itch sensitization. YKH showed approximately 200-fold inhibitory activity against NGF-induced neurite growth than that of neurotropin (positive control), a drug used clinically for treatment of chronic pruritus. Moreover, it also found that Uncaria hook, Bupleurum root and their chemical constituents rhynchophylline, hirsutine, and saikosaponin a, d showed inhibitory activities against NGF-induced neurite growth, suggesting they should mainly contribute to the inhibitory activity of YKH. Further study on the effects of YKH against epidermal nerve density in “itch-scratch” animal models is under investigation.

## 1. Introduction

Chronic pruritus is a major and distressing symptom of many cutaneous diseases such as in atopic dermatitis (AD), psoriasis, eczema, urticarial, and xerosis, which has a significant impact on quality of life for patients. In particular, daily pruritus is described in 87% to 91% of patients with AD [1,2], and reported a higher pruritus intensity compared to psoriasis patients [3]. Pruritus leads to an “itch-scratch” cycle that could bring about skin-barrier disruption, in turn, render the skin more permeable to irritants, allergens, and microorganisms. Thus, efforts for treatment of AD are directed to control the itching, suppress the inflammation, and restore the skin barrier [4]. Nevertheless, the treatment is still challenging, with no current universally accepted therapy for the itching, although some topical and systemic antipruritic drugs are available such as glucocorticoids and histamine H_1_ receptor antagonists [5].

On the other hand, complementary and alternative medicine is a conservative and increasingly popular approach to treat pruritus for both patients and medical providers [6], especially the traditional Chinese-Japanese medicines (also Kampo medicines). In recent years, a Kampo formula, yokukansankachimpihange (YKH) has been demonstrated to be effective in the treatment of itching of AD [7,8] or chronic urticaria and xerotic eczema patients [9] with psychoneurotic symptoms such as insomnia in Japan, however, few know about its pharmacological mechanism. Traditionally, YKH is used traditionally for treatment of neurasthenia, hysteria, insomnia, menopausal neurosis, and pediatric epilepsy [10], which consists of Atractylodes or Atractylodes lanceae rhizome, Angelica dahurica root, Bupleurum root, Citrus unshiu peel, Cnidium rhizome, Glycyrrhiza, Poria sclerotium, Pinellia tuber, and Uncaria hook.

The itch sensation is initiated by pruriceptive primary afferent neurons that convey information from the periphery to the CNS through the spinal cord, the cell bodies of primary afferent neurons are located in the dorsal root ganglia (DRG) and the trigeminal ganglion [11]. Although the pathophysiological mechanism of itch in AD is not fully clear, various peripheral and central mediators have been suggested to play a role in the pathophysiology of atopic eczema itch. Significant cross-talk occurs among stratum corneum, keratinocytes, immune cells, and nerve fibers [12]. The latter, in recent studies indicate that the density of epidermal sensory fibers is increased in AD and psoriasis, moreover, reduction in the fibers in animal models of AD and dry skin reduces scratching behavior, and thus suggesting that epidermal hyperinnervation is partly responsible for itch sensitization in cutaneous diseases. Furthermore, epidermal hyperinnervation is caused by an imbalance between the actions of nerve elongation factors (e.g., nerve growth factor, NFG) and repulsion factors (e.g., semaphorin 3A, Sema3A), and with respect to their role in detail has been well documented [13].

This study thus was focused on the inhibitory activity of YKH against NGF-induced neurite growth in cultured rat DRG neurons in an attempt to clarify its pharmacological actions, and to provide evidence that neurite growth inhibition contributes to better understanding YKH in anti-itching actions in comparison with a positive control neurotropin (NTP), a drug used clinically for treatment of chronic neuropathic pain and pruritus.

## 2. Results and Discussion

### 2.1. Inhibition of YKH against Neurite Length Induced with or without NGF and Cytotoxicity of Cultured DRG Neurons

**Figure 1:**
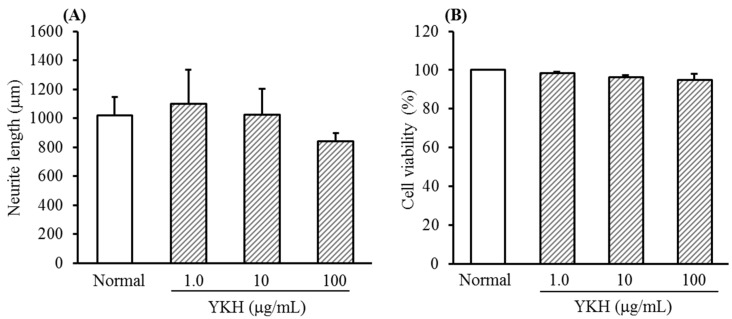
Changes of neurite length (**A**) and cell cytotoxicity (**B**) of yokukansankachimpihange (YKH) on cultured DRG neurons in absence of NGF. Values are the means ± S.E.M. of triplicate or quadruplet experiments.

YKH did not remarkably inhibit both neurite length (Figure 1A) and cell viability (Figure 1B) of cultured DRG neurons at a range of concentration from 1.0 to 100 μg/mL in the absence of NGF.

**Figure 2:**
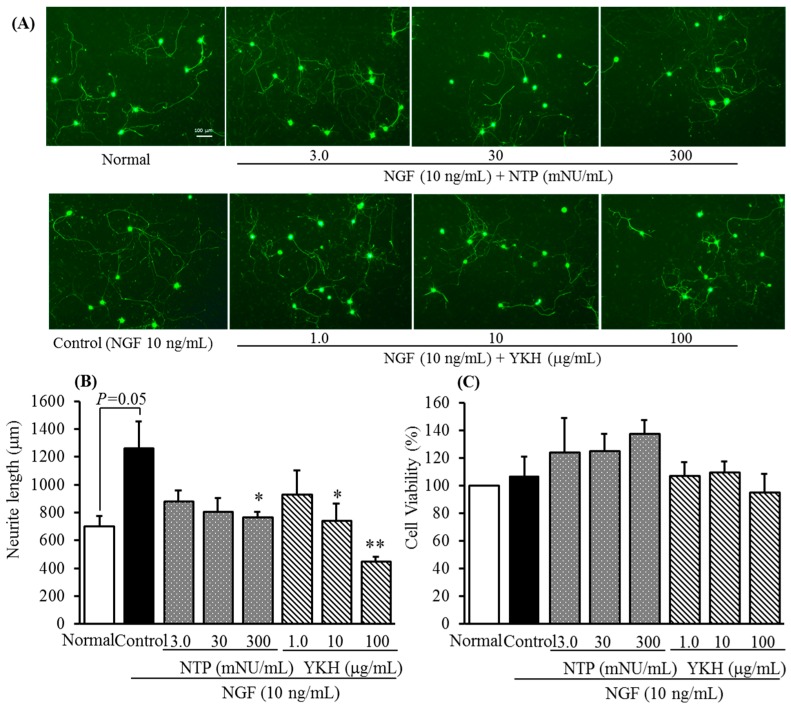
Inhibitory activities of Neurotropin^®^ (NTP, as a positive control) and yokukansankachimpihange (YKH) against NGF-induced neurite growth (**A**) representative images of DRG neurons from normal or NGF treated culture with test samples that showed peripherin immunofluorescence. Scale bar = 100 μm; (**B**) neurite elongation was calculated on the basis of length of all neurites) and cell cytotoxicity in cultured DRG neurons; (**C**). Values are the means ± S.E.M. of triplicate experiments. Normal *vs.* Control with Student’s *t*-test. * *p* < 0.05, ** *p* < 0.01 *vs.* Control (Dunnett’s test).

As shown in representative images of DRG neurons stained by peripherin (a sensory C fiber marker) immunofluorescence (Figure 2A), NGF significantly induced neurite growth of cultured DRG neurons for 48 h at a concentration 10 ng/mL than those cultured in the absence of NGF as a normal. It has been known that itch pathways are transmitted primarily via C-fibers [11,13]. Consistent with a published report [14], NTP, as a positive control, concentration-dependently inhibited the neurite growth induced by NGF at a range of concentration between 3.0 and 300 mNU/mL (Figure 2A). Meanwhile, no remarkable inhibitions against cell viability were observed in the treatment with NTP (Figure 2C).

In comparison with NTP, YKH concentration-dependently inhibited the neurite growth induced by NGF at a range of concentration from 1.0 to 100 μg/mL (Figure 2A,B) while did not affect cell viability of cultured DRG neurons (Figure 2C). In addition, YKH at a concentration 10 μg/mL showed approximately 200-fold inhibitory activity against NGF-induced neurite growth than that of NTP at a concentration 300 mNU/mL (about equal to 2000 μg/mL, calculated on the basis of recovery of freeze-dried powder weight, see Experimental Section 3.3.).

In fact, it is found not only to inhibit NGF-induced neurite growth of rat DRG neurons *in vitro* [14], but also to significantly reduce intraepidermal nerve growth in dry skin animal model [15], besides NTP has been used clinically for treatment of chronic neuropathic pain and pruritus.

### 2.2. Inhibitory Activities of Uncaria Hook and Bupleurum Root Together with Their Major Chemical Constituents against NGF-Induced Neurite Growth and Cytotoxicity of Cultured DRG Neurons

As described above, YKH consists of nine kinds of crude drugs, and we found that extracts of Uncaria hook ≥ Bupleurum root > Pinellia tuber showed inhibitory activities NGF-induced neurite growth of cultured DRG neurons without cytotoxicity at a concentration 100 μg/mL in a following screening assay in order to clarify which one is contribute to the inhibitory activity of YKH.

**Figure 3:**
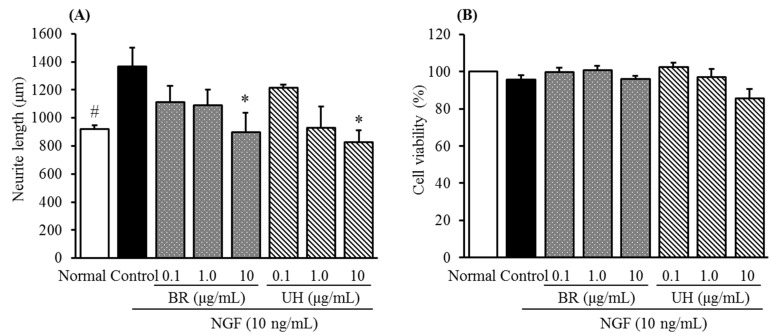
Inhibitory activities of Bupleurum Root (BR) and Uncaria Hook (UH) against NGF-induced neurite growth (**A**) and cell cytotoxicity in cultured DRG neurons (**B**). Values are the means ± S.E.M. of triplicate experiments. ^#^ *p* < 0.05 *vs.* Control (Student’s *t*-test); * *p* < 0.05 *vs.* Control (Dunnett’s test).

Here, the inhibitory activities of Bupleurum root and Uncaria hook together with their major chemical constituents were furthermore examined. As shown in Figure 3A,B, both the extracts of Bupleurum root and Uncaria hook showed concentration-dependent inhibitions without cell cytotoxicity of cultured DRG neurons at a range of concentration from 0.1 to 10 μg/mL, respectively. 

The compounds rhynchophylline, hirsutine, and saikosaponin a, d, which are not only exist largely but also usually used as chemical markers in the quality control of Uncaria hook and Bupleurum root, respectively. Therefore, they were employed to further the evaluation.

**Figure 4:**
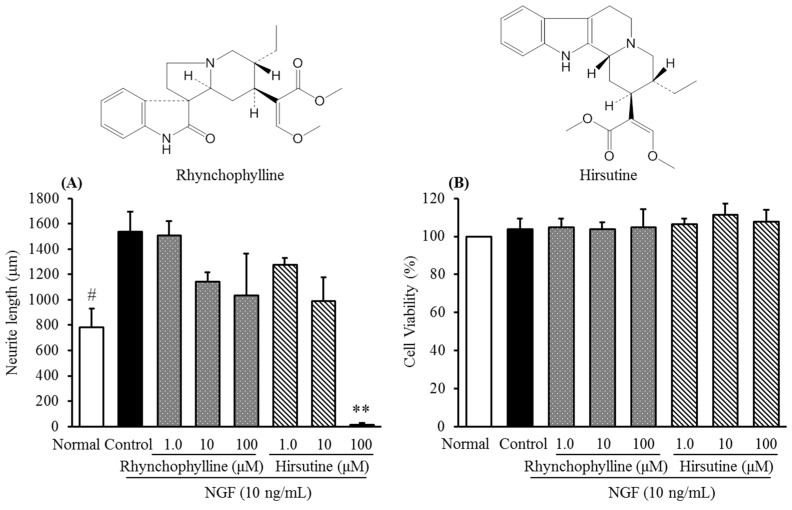
Chemical structures of rhynchophylline and hirsutine and their inhibitory activities against NGF-induced neurite growth (**A**) and cell cytotoxicity in cultured DRG neurons (**B**). Values are the means ± S.E.M. of triplicate experiments. ^#^*p* < 0.05 *vs.* Control (Student’s *t*-test); ** *p* < 0.01 *vs.* Control (Dunnett’s test).

Figure 4 shows the chemical structures of rhynchophylline and hirsutine in characteristic of indole alkaloid, and their inhibitory activities against NGF-induced neurite growth of cultured DRG neurons as well as cell viability. As shown in Figure 4A, both the compounds inhibited NGF-induced neurite growth with a concentration-dependent manner and did not significantly affect the cell viability of cultured DRG neurons at a range of concentration 1.0 to 100 μM. This result suggested that rhynchophylline and hirsutine could contribute to the inhibitory activity of Uncaria hook.

In addition, it should be noted that hirsutine showed a sharper inhibition at a concentration 100 μM than that of rhynchophylline although the reason is not clear, but at least suggests that the difference in their chemical structures may contribute to the different inhibitory activities.

Similar to rhynchophylline and hirsutine, saikosaponin a and d also showed inhibitory activities against NGF-induced neurite growth, however, cell viability of cultured DRG neurons were inhibited significantly at concentrations above 10 μM. Therefore, their inhibitory activities below a concentration of 10 μM, namely, at a range of concentration from 0.1–100 nM were further examined. 

**Figure 5:**
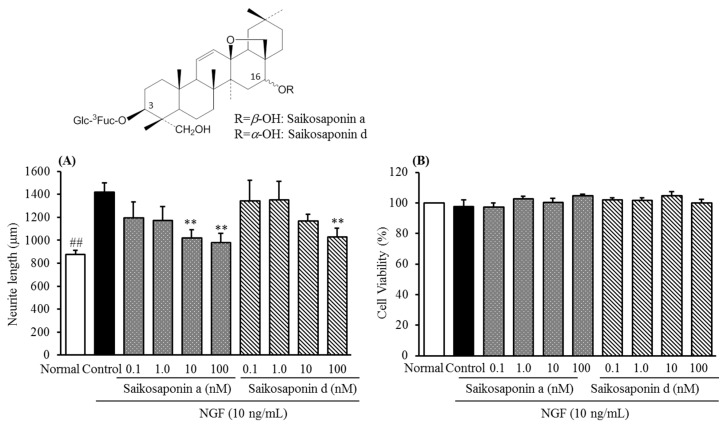
Chemical structures of saikosaponin a, d and their inhibitory activities against NGF-induced neurite growth (**A**) and cell cytotoxicity in cultured DRG neurons (**B**). Values are the means ± S.E.M. of triplicate or quadruplet experiments. ^##^ *p* < 0.01 *vs.* Control (Student’s *t*-test); ** *p* < 0.01 *vs.* Control (Dunnett’s test).

As shown in Figure 5, both saikosaponin a and d showed a concentration-dependent inhibition against NGF-induced neurite growth without significant cell toxicity of cultured DRG neurons. No significant difference in their structure-activity was observed in comparison with each other that implied the moiety at C-16 hydroxy group is not related to the activity. Interestingly, we found ginseng saponins (Rb_1_, Rg_1_, data not shown) do not have a concentration-dependent inhibition against NGF-induced neurite growth, although they have a similar chemical structures to saikosaponins.

Similarly, the chemical makers saikosaponin a and d could be contributed to the inhibitory activity of Bupleurum root as active principles.

Taken together, these results suggest that both Bupleurum root and Uncaria hook and their major chemical constituents should play a key role in the inhibitory activity of YKH against neurite growth.

## 3. Experimental Section

### 3.1. Experimental Animals

Male Sprague Dawley rats (five-to-eight weeks old) were purchased from SLC Inc. (Shizuoka, Japan). The animals were reared in an air-conditioned animal house facility (room temperature 23 ± 2 °C; 12 h light/dark cycle; relative humidity 55% ± 10%) at Kampo Research Laboratories in Kracie Pharma Ltd (Takaoka, Japan). The rats were housed in sterilized polypropylene cages and provided laboratory pellet chow (CE-2, Clea Japan Inc., Tokyo, Japan) and water *ad libitum*. Before experimental procedures, they were acclimated to the room for one week. This experiment was reviewed and approved by Experimental Animal Care Committee of Kracie Pharma Ltd. (Takaoka, Japan).

### 3.2. Plant Material and Extract Preparations

Information of plants’ materials including place of origin and batch Number used this study and the preparation method of dried extracts of YKH, and those crude drugs consisting YKH (Atractylodes or Atractylodes lanceae rhizome, Angelica dahurica root, Bupleurum root, Citrus unshiu peel, Cnidium rhizome, Glycyrrhiza, Poria sclerotium, Pinellia tuber and Uncaria hook) have been previously described in our previous study [16]. Dried extracts were dissolved in serum-free medium used in the following cell culture experiments.

### 3.3. Chemicals and Positive Control

In-house prepared standard references with purity 80%–100% rhynchophylline (Lot No. 156-1103A), hirsutine (Lot No. 157-1102) and saikosaponin a, d (Lot No. 0148, Lot No. 63-9306A) were dissolved in less than 0.1% of DMSO at final concentration and used for cell culture experiments. Neurotropin^®^ (NTP, 3.6 NU/3.0 mL, Lot No. 13109) injection was purchased from Nippon Zouki Pharmaceutical Company (Osaka, Japan), of which 150 mL was concentrated *in vacuo* and then freeze-dried to give 1.24 g of powder. The freeze-dried powder of NTP was then dissolved in phosphate buffered saline as a positive control before cell culture experiments, and added to the cultured DRG neurons at final concentrations ranging from 3.0 to 300 mNU/mL. NGF 2.5S (Nerve Growth Factor 2.5S subunit, native mouse protein, Lot No. 1577462) was purchased from Life technologies (Carlsbad, CA, USA).

### 3.4. Isolation and Culture of Rat DRG Neurons

Rat DRGs from the cervical to the lumbar level were harvest from young SD rats (6–9 weeks old) and cultured with or without treatment of NGF according to previously-established methods with some modification [17,18]. Briefly, DRGs were dissociated into a single-cell suspension with 0.2% collagenase type III (Worthington Biochemicals, Lot No. M3D14157, Lakewood, NJ, USA) at 37 °C for 2 h and centrifuged (×1000 rpm) at 4 °C for 5 min. The precipitation was washed twice with Hanks buffer solution (Nissui Pharmaceutical Co., Ltd, Lot No. 08820611, Tokyo, Japan) and cultured with 0.25% trypsin-EDTA (Life technologies, Lot No. 1227385) at 37 °C for 15 min. These ganglia were further triturated with fire-polished Pasteur pipettes after adding trypsin inhibitor (0.1 mg/mL, Life technologies, Lot No. 1183205), and then subjected to centrifugation (×1000 rpm, at 4 °C for 5 min) and washed the precipitation twice with Ham’s F12 medium containing 10% heat-inactivated fatal bovine serum (Biowest, Lot No. S10581S1820, Kansas, MO, USA). Finally, the washed precipitation was suspended into 10% FBS/F12 medium through 40 μm cell strainer at a density of 1.0 × 10^5^ cells/well (for NGF-induced neurite growth experiments) or 5.0 × 10^4^ cells/well (for cell survival experiments).

The cells were seeded on poly-D-Lysine coated culture 8-well chamber slides and cultured with 10% FBS/F12 medium and 1% penicillin/streptomycin for 24 h, and subjected to NGF-induced neurite growth experiments that the cells were cultured in serum-free medium (F12 medium with 2% B27 supplement; Life technologies, Lot No. 1480155) for 48 h in the absence or presence of NGF (10 ng/mL) together with or without test samples.

### 3.5. Neurite Growth Assessment and Cell Survival Assay

For neurite growth assessment, visualization of the neurites of cultured DRG neurons was performed using immunofluorescence [14,17]. Namely, the cells were fixed for 20 min in 4% paraformaldehyde (0.1 M phosphate buffer, pH 7.4), washed, and permeabilized with PBS containing 0.2% Triton X-100. And washed with PBS, blocked with 1% BSA–5% goat serum for 5 min and incubated for 2 h at room temperature in a 1:1000 mixture of rabbit polyclonal anti-peripherin (a sensory nerve marker, Abcam Plc, GR52209-1, Cambridge, UK), then washed with PBS again and added anti-rabbit IgG Alexa Fluor 488 for incubation (dilution 1:200) at room temperature for 45 min. The slides were mounted with Mount-Quick mounting medium and at least ten images of random 10× fields of DRG neurons were typically taken per slide by a fluorescence microscope (Leica DMI 4000 B, Leica Microsystems K.K, Tokyo, Japan). Length of the neurite growth in each image was analyzed using the Neuron J, a Java program for neurite tracing and quantification downloaded from website [19] which identifies cell bodies and traces all neurites associated with each cell body separately. The average extent of total outgrowth per slide was obtained from at least fifteen cells, and the final data were used for statistical analysis from triplicate or quadruplet experiments.

To assess cell survival of cultured DRG neurons, MTT assay was used and carried out according to the manufacturer’s specifications (MTT kit Lot No. GD089, Dojinddo Laboratories, Kumamoto, Japan). Briefly, cells were seeded on poly-D-Lysin coated 96-well culture plate and sub-cultured in serum free medium with or without test samples in the absence or presence of NGF (10 ng/mL) for 48 h. The culture medium in each well was discarded and replaced by MTT solution (0.5 mg/mL) in serum free medium and then incubated in a 37 °C humidified incubator in an atmosphere of 5% CO_2_ for 3 h. Finally, the absorbance of MTT formazan dissolved in DMSO was measured at a wavelength of 570 nm on a microplate reader (Multiskan™ Spectrum, Thermo Fisher Scientific Inc., Waltham, MA, USA). Absorbance values of non-treated cells were regards as 100% of cell viability.

### 3.6. Statistical Analysis

The experimental data are expressed as mean ± standard error of means (S.E.M). Student’s *t*-test was used for the comparison of the mean values between normal and control groups. Significant differences were determined by one-way analysis of variance (ANOVA) followed by Dunnett’s test for multiple comparisons. P values of less than 0.05 were considered statistically significant.

## 4. Conclusions

Chronic pruritus is a major and distressing symptom of many cutaneous diseases and remains a challenge in the clinic. Although the mechanisms which lead to pruritus and are not fully understood, recent advances have suggested that various peripheral and central mediators should play a role in the pathophysiology of pruritus. In particular, nerve density in the epidermis is partly responsible for itch sensitization in cutaneous diseases including AD, and Tominaga and colleagues [13] have well documented that epidermal innervation is regulated by the balance between nerve elongation and repulsion factors. 

As a representative nerve elongation factor, NGF occurs in higher levels not only in the lesional skin patients with AD, psoriasis, prurigo nodularis, contact dermatitis, and xerosis [20], but also in plasma of AD patients suggesting a useful marker in the diagnostic and treatment [21]. In this study, we examined inhibitory activities of YKH against nerve elongation, a Kampo formula is reported clinically to be effective in the treatment of pruritus, by means of NGF-induced neurite growth in cultured rat DRG neurons *in vitro*.

As a result, YKH showed a stronger inhibition against NGF-induced neurite growth than that of positive control NTP which used clinically for treatment of chronic neuropathic pain and pruritus in Japan. NTP has been found not only to inhibit NGF-induced neurite growth of rat DRG neurons *in vitro* [14], but also to significantly reduce intraepidermal nerve growth in a dry skin animal model [15]. From these facts it can be speculated that YKH may possess similar effects to NTP through reducing nerve elongation. In order to further verify this speculation, at present, a following animal study on the inhibitory effects of YKH against epidermal nerve density is under investigation with “itch-scratch” animal models. In fact, several of “itch-scratch” mouse models with intraepidermal neurite formation have been reported such as NC/Ng mice [22] and dry skin mice [23]. 

Furthermore, it also found that Uncaria hook, Bupleurum root and their chemical constituents rhynchophylline, hirsutine, and saikosaponin a, d showed inhibitory activities against NGF-induced neurite growth. This result suggests that both Bupleurum root and Uncaria hook and their major chemical constituents should be contributed mainly to the inhibitory activity of YKH against neurite growth. Interestingly, saikosaponin a, and Uncaria hook have been confirmed to have anti-inflammatory effects on picryl chloride-induced ear contact sensitivity in mice [24] and 2,4-dinitroflurorobenzene-induced AD-like dermatitis skin lesions in NC/Nga mice [25]. NGF and its receptor TrkA signaling pathways are essential for the neuron growth, specification and synapse formation. On the other hand, K-252a, a protein kinase inhibitor blocks NGF-induced neurite outgrowth by changing the tyrosine kinase phosphorylation [26]. Thus, in order to find out the mechinism of Uncaria hook, Bupleurum root and their active constituents, further study targeting at TrkA phosphorylation is under investigation.

In addition to the dermatologic and somatosensory aspects of pruritus, recently, the cognitive and emotional aspects in its central mechanism are notable and various pharmacotherapeutic agents used for depression and anxiety have been shown to be effective antipruritic medications such as selective serotonin reuptake inhibitors (SSRIs), noradrenergic selective serotoninergic antidepressant, and tricyclic antidepressamts [27]. Our previous study has actually found that YKH and its one of component Chimpi (Citrus unshiu peel) show antianxiety-like effects similar to that of fluoxetine belonging to SSRIs [16], suggesting that YKH should exert multiple pharmacological actions through peripheral and/or central pathways.
